# Low serum albumin: A significant predictor of reduced survival in patients with chronic heart failure

**DOI:** 10.1002/clc.23153

**Published:** 2019-02-07

**Authors:** Israel Gotsman, Ayelet Shauer, Donna R. Zwas, Ilgar Tahiroglu, Chaim Lotan, Andre Keren

**Affiliations:** ^1^ Heart Institute Hadassah University Hospital Jerusalem Israel; ^2^ Heart Failure Center Clalit Health Services Jerusalem Israel

**Keywords:** albumin, heart failure, outcome

## Abstract

**Background:**

Low serum albumin is common in patients with chronic heart failure (HF).

**Hypothesis:**

Albumin may have an impact on clinical outcome in HF. We evaluated the effect of albumin levels on clinical outcome in a real‐world cohort of patients with HF.

**Methods:**

All patients with HF at a health maintenance organization were followed for cardiac‐related hospitalizations and death.

**Results:**

A total of 5779 HF patients were included in the study; mean follow‐up was 576 days; median serum albumin was 4.0 g/dL (interquartile range 3.7‐4.2), and 12% of the patients had hypoalbuminemia (albumin<3.5 g/dL). Low albumin was associated with increasing age, higher urea and C‐reactive protein, lower sodium, hemoglobin, iron, less treatment with angiotensin‐converting enzyme inhibitor or angiotensin receptor blocker, reduced right ventricular function, and pulmonary hypertension. Cox regression analysis after adjustment for significant predictors demonstrated that decreasing quartiles of albumin was significantly associated with mortality: Lowest quartile compared to highest: hazard ratio (HR) 5.74, 95% confidence interval (CI) 4.08 to 8.07, *P* < 0.001. Cox regression analysis of albumin as a continuous parameter using restricted cubic splines after adjustment for significant parameters demonstrated that reduced albumin levels were directly associated with increased mortality (*P* < 0.001 for the adjusted model). Decreasing quartiles of albumin were also a significant predictor of increased cardiac‐related hospitalizations. A decrease in albumin on follow‐up was an independent predictor of increased mortality by Cox regression analysis: HR 2.58, 95% CI 2.12 to 3.14, *P* < 0.001.

**Conclusions:**

Low albumin provides important information regarding several detrimental processes in HF and is a significant predictor of a worse outcome in these patients.

## 1 BACKGROUND

Heart failure (HF) has emerged as a major epidemic and is a significant public health burden. It is associated with considerable morbidity and mortality.^1^ There are numerous clinical parameters that predict clinical outcome. Albumin, a standard clinical parameter that is associated with multiple parameters affecting outcome including nutritional, inflammatory, and volume status of HF patients, should have a significant impact on clinical outcome in these patients. Despite this, there is limited and conflicting evidence in the literature regarding this parameter as an important predictor of outcome in chronic HF.^2^, ^3^, ^4^ We hypothesized that this parameter would have a significant impact on clinical outcome in patients with chronic HF. We evaluated the impact of serum albumin levels on clinical outcome in a large real‐world cohort of patients with chronic HF.

## 2 METHODS

Clalit Health Services is the largest health maintenance organization (HMO) in Israel. It has a central computerized database in which all members have a complete digital record. The database includes demographics, comprehensive clinical data, diagnoses, and all laboratory data undertaken in a single centralized laboratory of the HMO. We identified and retrieved electronically from the computerized database all members with a diagnosis of HF as coded by the database in Jerusalem using the International Classification of Diseases, Ninth Revision (ICD‐9) code 428. About 6946 patients had a diagnosis of HF. Validation of the diagnosis of HF was performed on a randomly computer‐generated 5% of the diagnosed HF patients (N = 338) as previously described.^5^ Clinical parameters in this group of patients were statistically comparable with the whole HF cohort. We reviewed all available data from medical records and hospital admissions. In this group, 99% fulfilled the European Society of Cardiology (ESC) criteria for the diagnosis of HF^6^ based on typical symptoms, signs and structural or functional abnormalities of the heart per echocardiography. Only 1% had equivocal clinical data for the diagnosis of HF. Natriuretic peptides are not routinely performed in Israel and were not available for analysis. Serum albumin levels are routinely performed as part of a periodic work‐up in all members of the HMO. All patients in this cohort with serum albumin levels measured within 4 months of the time the database was established were included. About 84% of the patients (N = 5779) had an albumin level available for analysis. We also collected an additional albumin measurement, closest to the end of the study. Echocardiography data were available in digital form for analysis in 26% of the cohort (N = 1489). The clinical characteristics of these patients were very similar to the whole cohort. All echocardiography data including left ventricular ejection fraction, categorized into preserved (EF ≥ 50%) and reduced (EF < 50%) as well as measurements of dimensions were performed according to standard recommendations of the American Society of Echocardiography (ASE) and were acquired and verified by qualified personnel. All hospitalizations in cardiac and internal medicine departments including cardiac and internal intensive care units were retrieved and analyzed. Data on mortality were retrieved from the National Census Bureau. The Institutional Committee for Human Studies of Clalit Health Services approved the study protocol.

Biochemical analyses were performed at the HMO single centralized core laboratory with routine standardized methodologies on fresh samples of blood obtained after an overnight fast. Glucose levels were measured in plasma, and all other biochemical analyses were performed on serum. The laboratory is authorized to perform tests according to the international quality standard ISO‐9001. Normal levels for reference of serum albumin in the HMO laboratory are between 3.5 and 5 g/dL.

SPSS version 17.0 for Windows (SPSS Inc., Chicago, Illinois) and R Statistical Software version 3.0.1 for Windows (R Development Core Team) were used for the analyses. Comparison of the clinical characteristics was performed using the Mann‐Whitney *U* test for continuous variables and the ***χ*** ^**2**^ Test for categorical variables. Clinical predictors were transformed where appropriate. Log_10_ was used for logarithmic transformations with the exception of estimated glomerular filtration rate that a square root transformation was used. Follow‐up time was calculated using Kaplan‐Meier estimate of potential follow‐up.^7^ Kaplan‐Meier curves, with the log‐rank test, were used to compare survival according to albumin levels. Multivariate Cox proportional hazards regression analysis was used to evaluate independent variables that determined survival. Parameters included in the multivariate Cox regression analysis incorporated age, gender, and other clinically significant parameters as well as significant clinical and laboratory parameters on univariable analysis with the addition of significant drug therapy in separate models. Restricted cubic spline multivariable cox regression analysis was performed to evaluate the relationship between albumin as a continuous parameter and mortality. Proportionality assumptions of the Cox regression models were evaluated by log‐log survival curves and with the use of Schoenfeld residuals. An evaluation of the existence of confounding or interactive effects was made between variables and their possible collinearity. A *P* value of <0.05 was considered statistically significant.

## 3 RESULTS

### 3.1 Clinical parameters

The study cohort included 5779 HF patients. Supporting information Figure SS1 presents the distribution of serum albumin levels in the HF cohort. Mean albumin levels were 3.9 ± 0.43 g/dL; Median 4.0 g/dL (interquartile range [IQR] 3.7‐4.2 g/dL). About 12% of the patients (N = 704) had hypoalbuminemia (albumin<3.5 g/dL). The patient cohort was divided in quartiles according to albumin levels. Table 1 presents the demographics and clinical parameters of the patients stratified according to these quartiles. Low albumin was associated with increasing age, women gender, hypertension, peripheral vascular disease, prior stroke, dementia, and increased Charlson Comorbidity Index but less with ischemic heart disease. Low albumin was associated with lower body mass index, higher creatinine, and urea but lower sodium, hemoglobin, glucose, cholesterol, triglycerides, calcium, and iron. Low albumin was associated with increased C‐reactive protein. Patients with low albumin were treated less with angiotensin‐converting enzyme inhibitors (ACE‐Is) or angiotensin receptor blockers (ARBs), beta blockers and thiazides and more with furosemide. Low albumin was associated with preserved LV EF, smaller dimensions of the left ventricle, reduced RV systolic function, severe tricuspid regurgitation, and pulmonary hypertension. Multivariable linear regression demonstrated that older age, non‐ischemic heart disease, increased urea, low hemoglobin and sodium, non‐treatment with ACE‐I/ARB and thiazide, low iron and increased CRP were predictive of low serum albumin (*R* ^2^ = 0.278, *P* < 0.0001, Table SS1).

**Table 1:** Demographics and clinical characteristics of patients with heart failure according to albumin levels

	Albumin levels (g/dL)					*P* value
Variable	<3.8 (N = 1569)	3.8‐4 (N = 1690)	4.1‐4.2 (N = 1145)	>4.2 (N = 1375)	Total (N = 5779)	
Age (years)	80 ± 12	77 ± 12	74 ± 13	70 ± 14	75 ± 13	<0.001
Gender (male)	678 (43)	790 (47)	585 (51)	853 (62)	2906 (50)	<0.001
Diabetes mellitus	745 (47)	827 (49)	555 (48)	628 (46)	2755 (48)	0.31
Hypertension	1364 (87)	1428 (84)	945 (83)	1069 (78)	4806 (83)	<0.001
Hyperlipidemia	1179 (75)	1422 (84)	1006 (88)	1218 (89)	4825 (83)	<0.001
Ischemic heart disease	1082 (69)	1246 (74)	869 (76)	1073 (78)	4270 (74)	<0.001
Atrial fibrillation	399 (25)	465 (28)	319 (28)	329 (24)	1512 (26)	0.06
Prior coronary bypass surgery	200 (13)	270 (16)	228 (20)	289 (21)	987 (17)	<0.001
Chronic obstructive lung disease	129 (8)	193 (11)	137 (12)	125 (9)	584 (10)	0.002
Peripheral vascular disease	338 (22)	325 (19)	186 (16)	197 (14)	1046 (8)	<0.001
Prior stroke/transient ischemic attack	478 (30)	385 (23)	231 (20)	245 (18)	1339 (23)	<0.001
Dementia	290 (18)	166 (10)	73 (6)	68 (5)	597 (10)	<0.001
Charlson Comorbidity Index	6.0 (5.0‐7.0)	6.0 (5.0‐7.0)	6.0 (5.0‐7.0)	5.0 (5.0‐6.0)	6.0 (5.0‐7.0)	<0.001
Body mass index (kg/m^2^)	28 (25‐32)	29 (26‐33)	29 (26‐33)	29 (26‐31)	29 (26‐32)	<0.001
Pulse (beats per minute)	72 (64‐80)	72 (64‐80)	72 (64‐80)	70 (63‐80)	72 (64‐80)	0.08
Systolic blood pressure (mm Hg)	126 (115‐140)	129 (118‐140)	128 (117‐138)	127 (115‐137)	128 (117‐140)	0.04
Diastolic blood pressure (mm Hg)	70 (62‐78)	70 (64‐79)	70 (63‐79)	70 (65‐80)	70 (64‐79)	<0.001
Laboratory data						
Creatinine (mg/dL)	1.1 (0.8‐1.6)	1.0 (0.8‐1.4)	1.0 (0.8‐1.3)	1.0 (0.8‐1.2)	1.0 (0.8‐1.4)	<0.001
Estimated glomerular filtration rate (mL/min per 1.73 m^2^)^a^	54 (35‐78)	59 (42‐80)	64 (47‐81)	69 (52‐85)	61 (44‐81)	<0.001
Urea (mg/dL)	58 (41‐88)	50 (37‐71)	47 (36‐66)	43 (33‐57)	48 (36‐69)	<0.001
Sodium (mEq/L)	139 (137‐141)	140 (138‐142)	140 (138‐142)	140 (139‐142)	140 (138‐142)	<0.001
Hemoglobin (g/dL)	12 (11‐13)	13 (12‐14)	13 (12‐14)	14 (12‐15)	13 (12‐14)	<0.001
Albumin (g/dL)	3.5 (3.2‐3.6)	3.9 (3.8‐4.0)	4.1 (4.1‐4.2)	4.4 (4.3‐4.5)	4.0 (3.7‐4.2)	<0.001
Glucose (mg/dL)	98 (85‐130)	99 (88‐128)	102 (90‐128)	101 (91‐128)	100 (88‐128)	<0.001
Total cholesterol (mg/dL)	156 (132‐184)	162 (140‐190)	165 (142‐189)	167 (144‐194)	163 (140‐190)	<0.001
Low‐density lipoprotein (mg/dL)	88 (69‐110)	89 (72‐111)	90 (73‐110)	92 (74‐112)	90 (72‐112)	0.007
Triglycerides (mg/dL)	115 (86‐157)	122 (91‐166)	128 (96‐177)	131 (98‐187)	125 (92‐173)	<0.001
Calcium (mg/dL)	8.8 (8.5‐9.1)	9.1 (8.9‐9.4)	9.3 (9.0‐9.5)	9.4 (9.2‐9.7)	9.2 (8.8‐9.4)	<0.001
Iron (μg/dL)	49 (35‐65)	60 (46‐78)	65 (48‐85)	71 (54‐90)	59 (44‐79)	<0.001
C‐reactive protein (mg/dL)	1.2 (0.5‐3.2)	0.7 (0.3‐1.6)	0.6 (0.3‐1.3)	0.4 (0.2‐0.9)	0.7 (0.3‐1.6)	<0.001
Medication						
ACE‐I/ARB	1063 (68)	1311 (78)	909 (79)	1093 (79)	4376 (76)	<0.001
Beta blockers	997 (64)	1154 (68)	823 (72)	983 (71)	3957 (68)	<0.001
Spironolactone	519 (33)	558 (33)	387 (34)	421 (31)	1885 (33)	0.32
Furosemide	1145 (73)	1229 (73)	758 (66)	772 (56)	3904 (68)	<0.001
Thiazide	287 (18)	433 (26)	340 (30)	349 (25)	1409 (24)	<0.001
Digoxin	173 (11)	182 (11)	126 (11)	138 (10)	619 (11)	0.82

	Albumin levels (g/dL)					*P* value
Echocardiography data	<3.8 (N = 455)	3.8‐4 (N = 428)	4.1‐4.2 (N = 299)	>4.2 (N = 307)	Total (N = 1489)	
Left ventricle ejection fraction (<50%)	235 (52)	221 (52)	172 (58)	188 (61)	816 (55)	0.02
Ejection fraction (%)	51 (34‐61)	49 (35‐61)	47 (33‐60)	46 (31‐58)	48 (33‐60)	0.10
Left ventricle end‐diastolic diameter (cm)	5.3 (4.7‐6.0)	5.3 (4.8‐6.0)	5.4 (4.9‐6.0)	5.5 (5.0‐6.0)	5.4 (4.8‐6.0)	0.02
Reduced right ventricle systolic function^b^	203 (45)	168 (40)	86 (30)	118 (39)	575 (39)	<0.001
Peak tricuspid regurgitation gradient (mm Hg)	35 (29‐44)	34 (28‐43)	32 (25‐41)	32 (24‐42)	34 (27‐43)	0.002

Abbreviations: ACE‐I, angiotensin‐converting enzyme inhibitor; ARB, angiotensin receptor blocker.

Data are presented as median (inter‐quartile range) for continuous variables and counts (percentages) for categorical variables. *P* value by the Kruskal‐Wallis Test for continuous variables and the *χ* ^2^ test for categorical variables.

Diabetes mellitus defined as fasting plasma glucose ≥126 mg/dL or glucose lowering treatment, hypertension as blood pressure > 140/90 mm Hg measured on several occasions or anti‐hypertensive treatment and hyperlipidemia as low‐density lipoprotein >130 mg/dL, fasting serum triglycerides >200 mg/dL, or lipid lowering treatment.

a Estimated glomerular filtration rate was calculated using the modified Modification of Diet in Renal Disease (MDRD) Equation (175 * serum creatinine^−1.154^ * age^−0.203^. For females a correction factor is used multiplying by 0.742).

b Defined as qualitative systolic ventricular function less than normal.

### 3.2 Albumin levels and clinical outcome

**Figure 1:**
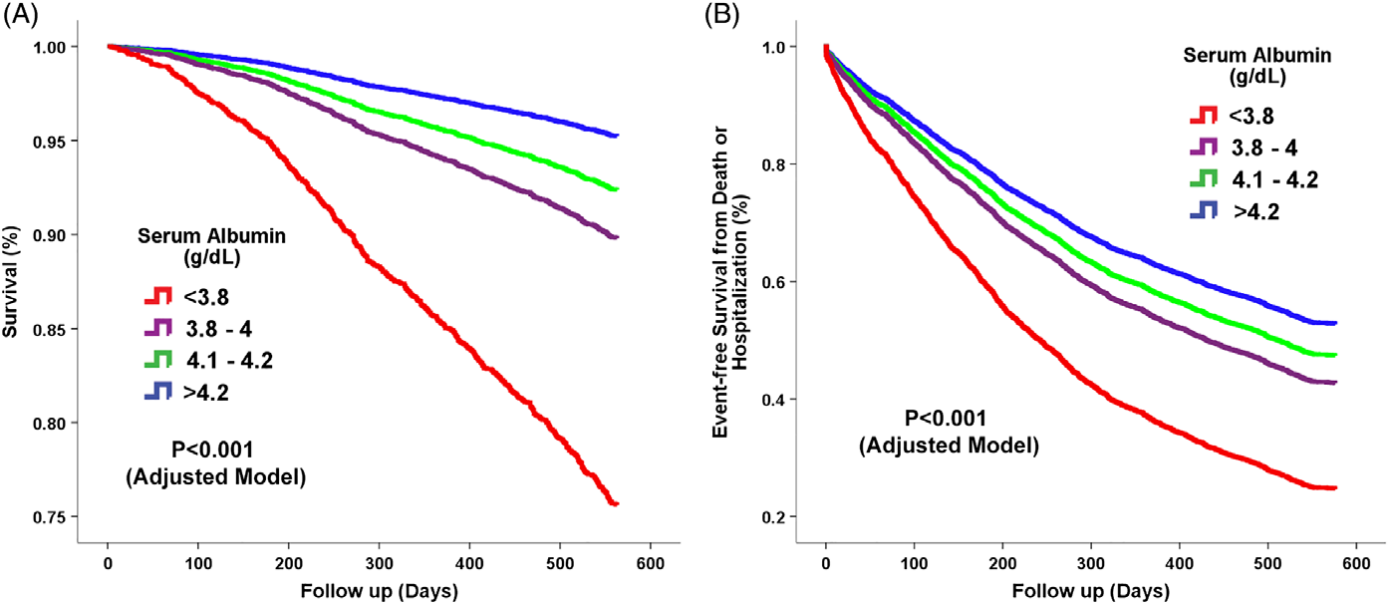
Cox regression adjusted survival plots according to serum albumin levels. A, Decreasing albumin levels were directly associated with reduced survival, *P* < 0.001 for the adjusted model. B, Decreasing albumin levels were directly associated with decreased event‐free survival from death or cardiovascular‐hospitalizations, *P* < 0.001 for the adjusted model. Variables included in the adjusted model included age, gender, ischemic heart disease, diabetes, hyperlipdemia, hypertension, atrial fibrillation, log‐transformed body mass index, log‐transformed pulse, log‐transformed serum urea levels, square root‐transformed estimated glomerular filtration rate, hemoglobin, and serum sodium

**Figure 2:**
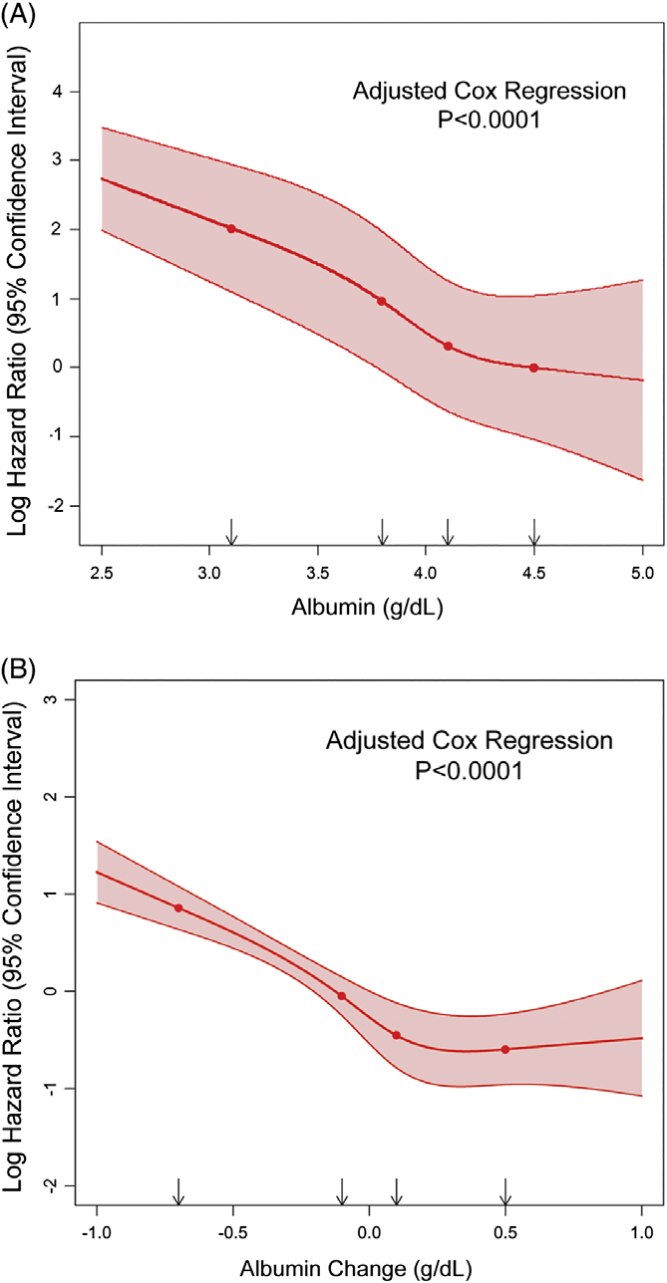
Mortality as a function of serum albumin and change in serum albumin. A, Cox regression analysis with adjusted hazard ratio for mortality (with 95% [CI]) of serum albumin as a continuous variable using restricted cubic splines with 4 knots at the 5th, 35th, 65th and 95th percentiles of serum albumin (3.1, 3.8, 4.1, 4.5 g/dL), *P* < 0.0001 for the adjusted model. Variables included in the model included age, gender, ischemic heart disease, diabetes, hyperlipdemia, hypertension, atrial fibrillation, log‐transformed body mass index, log‐transformed pulse, log‐transformed serum urea levels, square root‐transformed estimated glomerular filtration rate, hemoglobin, and serum sodium. B, Cox regression analysis with adjusted hazard ratio for mortality (with 95% CI) of the change in serum albumin as a continuous variable using restricted cubic splines with knots at the 5th, 35th, 65th, and 95th percentiles of serum albumin change (−0.7, −0.1, 0.1, 0.5 g/dL), *P* < 0.0001 for the adjusted model. Variables included in the model included age, gender, ischemic heart disease, diabetes, hyperlipdemia, hypertension, atrial fibrillation, log‐transformed body mass index, log‐transformed pulse, log‐transformed serum urea levels, square root‐transformed estimated glomerular filtration rate, hemoglobin, serum sodium, and baseline albumin

The median follow‐up period was 576 days. The overall mortality rate during this period was 14%. Survival rate by Kaplan‐Meier analysis demonstrated that decreasing albumin levels were directly associated with reduced survival (decreasing albumin quartiles: 95.7 ± 0.6% vs 92.9 ± 0.8% vs 88.3 ± 0.8% vs 67.2 ± 1.2%, *P* < 0.001). Decreasing albumin levels were also directly associated with decreased event‐free survival from death or cardiovascular‐hospitalizations (decreasing albumin quartiles: 58.6 ± 1.3% vs 52.0 ± 1.5% vs 42.9 ± 1.2% vs 23.4 ± 1.1%, *P* < 0.001). Multivariable Cox regression analysis after adjustment for significant predictors demonstrated that decreasing albumin levels were a significant predictor of mortality (Table 2 and Figure 1A). After adjustment for other significant predictors (see Table 2 for significant clinical predictors included), low albumin levels (<3.8 g/dL) was associated with a 5‐fold increase in mortality compared to the highest albumin quartile (>4.2 g/dL), with a hazard ratio of 5.74, 95% confidence interval (CI) 4.08 to 8.07, *P* < 0.001. Inclusion of HF medications (Table 3) demonstrated a very similar result with a direct relation between reduced albumin levels and mortality. We performed a further sensitivity analysis by analyzing albumin levels as a continuous parameter using restricted cubic splines. Cox regression analysis after adjustment for significant parameters included in Table 2 demonstrated a direct inverse relationship between albumin and mortality (Figure 2A). This analysis demonstrated that any decrease in albumin was a predictor of mortality with a continuous increase in the risk with lower albumin levels (Figure 2A), *P* < 0.0001 for the adjusted model.

**Table 2:** Predictors of mortality by Cox regression analysis

Variable	Univariable		Multivariable	
	Hazard ratio (95% CI)	*P* value	Hazard ratio (95% CI)	*P* value
Age (years)	1.04 (1.04‐1.05)	<0.001	1.02 (1.02‐1.03)	<0.001
Gender (male)	0.89 (0.78‐1.02)	0.09	1.14 (0.96‐1.34)	0.12
Diabetes mellitus	1.06 (0.93‐1.21)	0.40	1.09 (0.92‐1.29)	0.35
Hypertension	1.46 (1.19‐1.79)	<0.001	1.19 (0.92‐1.54)	0.19
Hyperlipidemia	0.56 (0.48‐0.65)	<0.001	0.76 (0.62‐0.93)	0.008
Ischemic heart disease	1.02 (0.87‐1.19)	0.80	1.05 (0.87‐1.28)	0.59
Atrial fibrillation	0.97 (0.83‐1.13)	0.68	0.88 (0.74‐1.05)	0.16
Body mass index^a^ (kg/m^2^)	0.15 (0.06‐0.38)	<0.001	0.32 (0.11‐0.94)	0.04
Pulse^a^ (beats per minute)	3.20 (1.41‐7.27)	0.01	3.14 (1.34‐7.36)	0.008
Urea (mg/dL)^a^	8.96 (6.73‐11.91)	<0.001	5.44 (3.16‐9.38)	<0.001
eGFR^b^ (mL/min per 1.73 m^2^)	0.85 (0.82‐0.88)	<0.001	1.05 (0.99‐1.12)	0.08
Hemoglobin (g/dL)	0.74 (0.71‐0.77)	<0.001	0.92 (0.87‐0.97)	0.001
Sodium (mEq/L)	0.92 (0.90‐0.93)	<0.001	0.97 (0.95‐1.00)	0.02
Albumin levels (g/dL)		<0.001		<0.001
>4.2	1.0 (Reference)		1.0 (Reference)	
4.1‐4.2	1.67 (1.19‐2.34)	0.003	1.63 (1.09‐2.43)	0.02
3.8‐4	2.81 (2.10‐3.76)	<0.001	2.21 (1.55‐3.14)	<0.001
<3.8	9.19 (7.02‐12.03)	<0.001	5.74 (4.08‐8.07)	<0.001

Abbreviation: eGFR, estimated glomerular filtration rate.

Data are presented as hazard ratio (95% confidence interval), *P* value.

a Log‐transformed.

b Square root‐transformed.

**Table 3:** Hazard ratio for clinical outcome according to albumin levels by Cox regression analysis

Clinical parameter	Albumin levels (g/dL)				*P*‐value
	>4.2	4.1‐4.2	3.8‐4	<3.8	
Death					
Univariable	1.0 (Reference)	1.67 (1.19‐2.34), 0.003	2.81 (2.10‐3.76), <0.001	9.19 (7.02‐12.03), <0.001	<0.001
Multivariable	1.0 (Reference)	1.63 (1.09‐2.43), 0.02	2.21 (1.55‐3.14), <0.001	5.74 (4.08‐8.07), <0.001	<0.001
Multivariable and drugs	1.0 (Reference)	1.66 (1.11‐2.47), 0.01	2.20 (1.54‐3.14), <0.001	5.48 (3.89‐7.71), <0.001	<0.001
Death and cardiovascular hospitalization					
Univariable	1.0 (Reference)	1.23 (1.10‐1.39), <0.001	1.60 (1.44‐1.78), <0.001	2.84 (2.57‐3.14), <0.001	<0.001
Multivariable	1.0 (Reference)	1.17 (1.02‐1.34), 0.03	1.33 (1.18‐1.51), <0.001	2.19 (1.93‐2.48), <0.001	<0.001
Multivariable and drugs	1.0 (Reference)	1.14 (1.00‐1.31), 0.06	1.30 (1.15‐1.47), <0.001	2.17 (1.91‐2.46), <0.001	<0.001

Data are presented as hazard ratio (95% confidence interval), *P* value.

Parameters that were included in the multivariable analysis model were age, gender, ischemic heart disease, diabetes, hyperlipidemia, hypertension, atrial fibrillation, log‐transformed body mass index, log‐transformed pulse, log‐transformed serum urea levels, square root‐transformed estimated glomerular filtration rate, hemoglobin, and serum sodium.

Parameters that were included in the multivariable and drugs analysis included the above parameters and the drug treatment with angiotensin‐converting enzyme inhibitor/angiotensin receptor blocker, beta blocker, furosemide, spironolactone, thiazide, and digoxin.

Low albumin levels were also a significant predictor of the combined endpoint of death and cardiovascular hospitalization. Multivariate Cox regression analysis after adjustment for significant predictors demonstrated that decreasing albumin levels were a significant predictor of the combined endpoint (Table 3 and Figure 1B). After adjustment for other significant predictors, low albumin levels (<3.8 g/dL) was associated with a 2‐fold increase in death and cardiovascular hospitalization compared with the highest albumin quartile (>4.2 g/dL), with a hazard ratio of 2.19, 95% CI 1.93 to 2.48, *P* < 0.001. Inclusion of HF medications (Table 3) demonstrated a very similar result, with a direct relation between reduced albumin levels and the combined endpoint of mortality and cardiovascular hospitalization Table 3.

Subgroup analysis of patients with echocardiographic data regarding left ventricular EF (N = 1489) revealed a very similar result, with decreasing levels of albumin a significant independent predictor of death or the combined endpoint of death and cardiovascular hospitalization after adjustment for significant predictors including LV EF. This was also evident in the model that included medications. Separate analysis of patients with preserved vs reduced LV EF demonstrated that these results were equally evident in both groups. In addition, in the Cox regression models of the entire cohort, there was no significant interaction between albumin and LV EF. These results suggest that low albumin is a significant predictor of clinical outcome in HF with preserved as well as reduced LV EF.

### 3.3 Changes in albumin and impact on outcome

Follow‐up albumin measurement was available in 4452 of the patients (77% of the cohort). The median difference between the first and follow‐up albumin was 335 days (IQR 212‐433). The mean change in albumin levels was −0.05 ± 0.38 g/dL with a median of 0.0 g/dL (IQR [−0.2 to 0.2]. About 46% of the patients (2043) had a decrease in albumin over time with a median decrease of (−0.3) g/dL (IQR [−0.5 to −0.1]. Decrease in albumin on follow‐up was a significant predictor of increased mortality by Cox regression analysis after adjustment for significant predictors including medications and baseline albumin; HR 2.58, 95% CI 2.12‐3.14, *P* < 0.001. A sensitivity analysis of the change in albumin levels as a continuous parameter using restricted cubic splines was performed. Cox regression analysis after adjustment for significant parameters including baseline albumin demonstrated a direct relationship between the decrease in albumin and mortality (Figure 2B). This analysis demonstrated that any decrease in albumin over time was a predictor of mortality with a continuous increase in the risk with decreasing albumin levels on follow‐up (Figure 2B), *P* < 0.0001 for the adjusted model.

## 4 DISCUSSION

In this cohort of real‐world HF patients, low albumin level was a significant predictor of death as well as death and cardiovascular hospitalizations. This direct inverse relationship between albumin and clinical outcome appears to be relevant across the whole spectrum of albumin levels. Moreover, the present study suggests that a decrease in albumin over time conveys additional predictive value with a significant negative impact on clinical outcome.

Albumin is a hepatic synthesized protein that is associated with numerous detrimental biological processes that are present in HF and pertain to a worse outcome.^9^ Low albumin in HF patients may be due to volume overload causing hemodilution, a chronic inflammatory state, liver congestion, malnutrition and cachexia, all causing decreased synthesis of albumin, and less commonly, protein loss due to proteinuria or enteropathy. Conversely, low albumin has a negative impact on several clinical aspects of HF. Albumin has an important role in numerous physiological processes including maintaining colloid osmotic pressure and microvascular integrity, ligand‐binding and transport of substances, antioxidant and antithrombotic functions, and enzymatic activities.^10^ Low albumin in HF promotes and aggravates congestion due to reduced intravascular colloid osmotic pressures,^11^ increases oxidative stress,^12^ inflammation,^10^ and the susceptibility to infections. Therefore, low albumin is a summation of numerous deleterious factors in HF patients and would be expected to give important prognostic information in HF. Despite this, there is sparse data in the literature regarding this parameter as a prognostic factor in chronic HF.

In the setting of acute HF, albumin has shown to have an important impact on survival and several papers demonstrate that albumin is an important prognostic parameter of clinical outcome.^8^, ^13^, ^14^, ^15^, ^16^, ^17^, ^18^ Interestingly, albumin was not helpful in guiding diuretic therapy or predicting short‐term outcome in acute HF diuretic therapy trials.^19^ In the setting of chronic HF, data are much less abundant and less conclusive; a study on chronic HF patients with reduced EF referred to an academic hospital‐based HF center^2^ demonstrated that hypoalbuminemia was a significant predictor of survival. This was also shown in HF with preserved EF,^3^ although the study targeted hospitalized patients. In contrast, albumin was not found to be a predictor of clinical outcome in a substudy of the CHARM program after adjustment for bilirubin levels.^4^ This divergence may be related to the different study populations in randomized studies which enroll younger and predominantly male subjects vs real‐world cohort studies that include older and a higher percentage of female patients. This was the case in the study of HF with preserved EF^3^ and in the present study. However, there was no interaction between age and gender with albumin on prediction of outcome in our study. The present study provides additional evidence to support the importance of albumin in risk stratification in patients with chronic HF. In the present study, low albumin and not only hypoalbuminemia was a strong predictor of reduced survival and increased hospitalizations. This was evident in patients with reduced EF as well as in patients with preserved EF.

The prevalence of hypoalbuminemia in HF patients in this study was 12%. This is lower than previously published data, with a prevalence of 18% or higher.^2^, ^3^, ^4^ This could be due the fact that the present cohort was a real‐world community‐based study which represents a broader spectrum of stable HF patients and thus perhaps represents the real‐world prevalence of hypoalbuminemia in HF.

In the present study, low albumin was associated with increasing age, lower kidney function, lower hemoglobin, sodium and iron but less with ACE‐I/ARB treatment. It was associated with increased C‐reactive protein but not with body mass index. This suggests that the nutritional state is perhaps not the dominant reason for reduced albumin in this HF cohort and other reasons such as inflammation may be more important. This was also seen in a previous study.^2^ The minimal association of albumin with body mass index could be related to the fact that reduction in albumin occurs only at a more advanced stage of nutritional deficiency and reduction in weight and is largely affected by other factors such as inflammation. However, it is possible that body mass that is derived from body weight is not accurate enough in HF to estimate dry body (muscle) weight, which is the best measure of nutritional state. Low albumin was also associated with right HF, tricuspid regurgitation, and pulmonary hypertension. This most probably is related to the increased liver congestion seen in these patients,^20^ suppressing albumin synthesis.

An interesting and important finding in the present study was that changes in albumin levels over time have prognostic implications in chronic HF. A decline in albumin levels predicted a worse clinical outcome independently of baseline albumin levels. A very similar finding was reported in the setting of chronic HF.^21^ This finding is plausible biologically, as decline in albumin over time characterizes the deteriorating state of the HF patient. Acute temporal changes in albumin have been analyzed in the setting of acute HF^16^, ^22^ and provide prognostic information. An increase in albumin levels during hospitalization predicted a better long‐term outcome, while a decrease predicted a worse outcome. In the acute setting, these changes are related to the acute event severity, sequel and its progression. In the chronic setting, these changes probably reflect the progression of the chronic disease with albumin reflecting the detrimental biological processes associated with low albumin and its significance to prognosis.

These findings of a prognostic importance of albumin raise the question regarding targeting albumin as a therapeutic avenue. Assessing albumin levels in HF patients is obviously important to evaluate the general status of the patient and provide prognostic information, as demonstrated in the present study. Albumin levels can help guide the necessity of further work‐up regarding the inflammatory, nutritional, and volume status of the chronic patient. Increasing albumin by appropriate nutrition is relevant in patients with malnutrition. Closer follow‐up and additional optimization of medical therapy may be warranted in all patients with low albumin. The benefit of increasing albumin in the chronic setting by artificial methods like protein supplements or albumin has not been evaluated.

Limitations of this study: The present study was an observational study. Data regarding clinical parameters and drug therapy was based on a digitized database. Although this database was validated and found to be highly accurate, not all data could be verified. While we tried to adjust for clinical relevant parameters, not all clinical parameters were available and it is impossible to adjust for all variables that may affect outcome. In particular, data on functional capacity and natriuretic peptide levels were not available. In addition, the cohort was a community‐based cohort and the findings may not be applicable in more advanced or hospital‐based HF cohorts.

In conclusion, low serum albumin provides important information regarding several detrimental processes in HF and is a significant predictor of a worse outcome in these patients. A decline in albumin was also a predictor of reduced survival.

## CONFLICTS OF INTEREST

The authors have no conflicts of interest to report.

## Supporting information


**Figure S1.** Histogram of serum albumin levels in the heart failure cohort.

**Table S1.** Multivariable linear regression for prediction of increased serum albumin.
