# Supplementary figures and images for: Low serum albumin: A significant predictor of reduced survival in patients with chronic heart failure
Source: Clin Cardiol. 2019 Feb 7;42(3):365–72. doi: 10.1002/clc.23153 (PMC6712335; doi:10.1002/clc.23153)

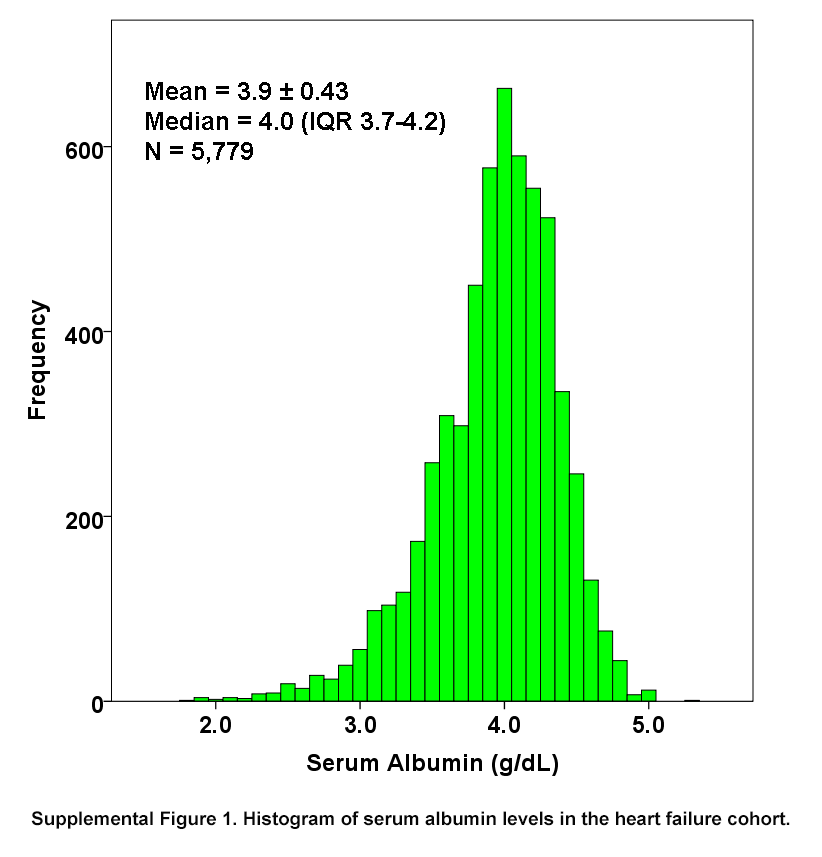

Supplement: Supplementary file 1 — Figure S1. Histogram of serum albumin levels in the heart failure cohort. [file CLC-42-365-s001.tif]
