# Supplementary material for: Low serum albumin: A significant predictor of reduced survival in patients with chronic heart failure
Source: Clin Cardiol. 2019 Feb 7;42(3):365–72. doi: 10.1002/clc.23153 (PMC6712335; doi:10.1002/clc.23153)
Supplement: Supplementary file 2 — Table S1. Multivariable linear regression for prediction of increased serum albumin. [file CLC-42-365-s002.docx]

Supplemental Table 1. Multivariable linear regression for prediction of increased serum albumin.

|  | Standardized Coefficients (β) | P Value |
| --- | --- | --- |
| Age (years) | -0.206 | <0.001 |
| Gender (Male) | -0.008 | 0.78 |
| Ischemic Heart Disease | 0.067 | 0.02 |
| Body Mass Index (kg/m^2^)* | -0.023 | 0.41 |
| Pulse (beats per minute)* | -0.048 | 0.87 |
| Urea (mg/dL)* | -0.070 | 0.02 |
| Hemoglobin (g/dL) | 0.268 | <0.001 |
| Sodium (mEq/L) | 0.078 | <0.001 |
| ACE-inhibitor / ARB | 0.105 | <0.001 |
| Thiazide | 0.060 | 0.03 |
| Iron (µg/dL) | 0.153 | <0.001 |
| Total cholesterol (mg/dL) | 0.045 | 0.12 |
| C-Reactive Protein (mg/dL) | -0.120 | <0.001 |

Parameters in included in the linear regression included age, male gender, ischemic heart disease, log-transformed body mass index, log-transformed heart rate, log-transformed urea, hemoglobin, sodium, treatment with angiotensin-converting enzyme inhibitor/ angiotensin receptor blocker, thiazide, iron, total cholesterol, C-reactive protein. R^2^=0.278 for the adjusted model, P<0.0001

* Log-transformed
